# Clinical Features and Outcomes of Gallbladder Polyps in Children

**DOI:** 10.5152/tjg.2022.21944

**Published:** 2022-09-01

**Authors:** Orxan Ferzeliyev1, Berna Oğuz2, Tutku Soyer1, Özlem Boybey Türer1, Mithat Haliloglu2, Feridun Cahit Tanyel1

**Affiliations:** 1 1Departments of Pediatric Surgery, Hacettepe University Faculty of Medicine, Ankara, Turkey; 2 2Departments of Pediatric Radiology, Hacettepe University Faculty of Medicine, Ankara, Turkey

**Keywords:** Children, cholecystectomy, gallbladder polyps, hyperplastic polyp

## Abstract

**Background:**

Gallbladder polyps are rare lesions protruding into the gallbladder lumen with variable clinical presentation. No standard treatment algorithm has been developed for pediatric gallbladders, and the malignant potential of the gallbladder is not clear in children. Therefore, a retrospective study was performed to evaluate the clinical features and treatment options of gallbladder polyps in children.

**Methods:**

Between 2014 and 2020, children diagnosed with gallbladder polyps were evaluated for age, gender, clinical features, results of follow-up with ultrasound findings, and treatment options retrospectively.

**Results:**

The records of 15 patients with a mean age of 13.2 years (2-20 years) were included. The male: female ratio was 7 : 8. Gallbladder polyps was detected incidentally in 73.3% (n = 11) of the patients. Four (20%) of the patients were symptomatic (26.7%) and complained about abdominal pain. Laboratory tests were normal except in 3 patients who showed slightly increased liver function tests. Two of the patients had 3 polyps on admission. The polyps were 2-10 mm in size. The size of the polyp increased in 5 patients (33.3%) and disappeared in 4 patients (26.6%) in follow-up ultrasound examinations. Five of the patients underwent cholecystectomy and 1 of them was scheduled for surgery. Five of the asymptomatic patients who have polyps less than 10 mm in size are still on follow-up. In patients with cholecystectomy, the histopathology of gallbladders revealed cholesterol polyps (n = 2) and hyperplastic polyps (n = 2). One of the patients with cholecystectomy showed no polyps in histopathological evaluation.

**Conclusion:**

: Despite the lack of a standardized algorithm, our data suggested that multiple polyps, polyps with increased in size or greater than 10 mm, and the presence of symptoms might require cholecystectomy in children. Asymptomatic patients with small-sized polyps can be identified using ultrasound, and the polyps may disappear during the subsequent follow-up.

**Main Points**

- Gallbladder polyps are very rare in children and no evidence-based treatment algorithm has been developed for pediatric population.
- Multiple polyps, polyps with increased in size or greater than 10 mm, and the presence of symptoms might require cholecystectomy in children.
- Asymptomatic patients with small-sized polyps can be followed up with ultrasound and the polyps may disappear during the follow-up. Therefore, repeated ultrasonography is mandatory to avoid unnecessary cholecystectomy in children.

## Introduction

Gallbladder polyps (GP) are rare lesions protruding into the gallbladder lumen resulting from the proliferation of biliary mucosa.^1^ The widespread use of high-quality ultrasonography (US) leads to an increase in the detection of GP in both symptomatic and asymptomatic patients.^2^ The incidence of GP was reported to be 4%-7% in patients undergoing US.^3^ Christensen et al.^4^ classified GP as benign tumors, pseudotumors, and malignant neoplasms. According to the associated anomalies, some authors classified GP with associated anomalies as either primary or secondary.^2^ In the adult population, polyps larger than 10 mm, sessile and solitary polyps with irregular borders, associated gallstones, irregularity and thickening of gall bladder wall adjacent to polyps, and high growth rate during the follow-up are considered as risk factors for malignant transformation.^5,6^ However, no criteria for malignant transformation have been defined in children.

The prevalence of GP among children has not been reported. Similar to an adult’s series, GP may show different clinical courses during childhood. It has been reported that pediatric GPs may disappear during observational follow-up or may not be detected in the histological evaluation after cholecystectomy.^7,8^ Therefore, the natural history of pediatric GP show wide variation in children and little is known about their malignant potential. The treatment options of pediatric GP are extrapolated from adult series and no clear treatment algorithm has been developed for children. A retrospective study is performed to evaluate the clinical features and outcome of GP in children.

## Materials and Methods

The children diagnosed with GP, between 2014 and 2020, are included in the study. The medical records of the patients were evaluated for age, gender, clinical features, results of follow-up with US findings, and treatment options retrospectively. Children younger than 18 years of age on admission were included. Abdominal US was performed with a Philips Affiniti 70G (Philips, the Netherlands) and Siemens Acuson S3000 (Siemens, Erlangen, Germany) ultrasound machines using a curved transducer (frequency range: 6-1 MHz). Local Ethical Committee (2020/14) approved the study.

## Results

**Figure 1.**
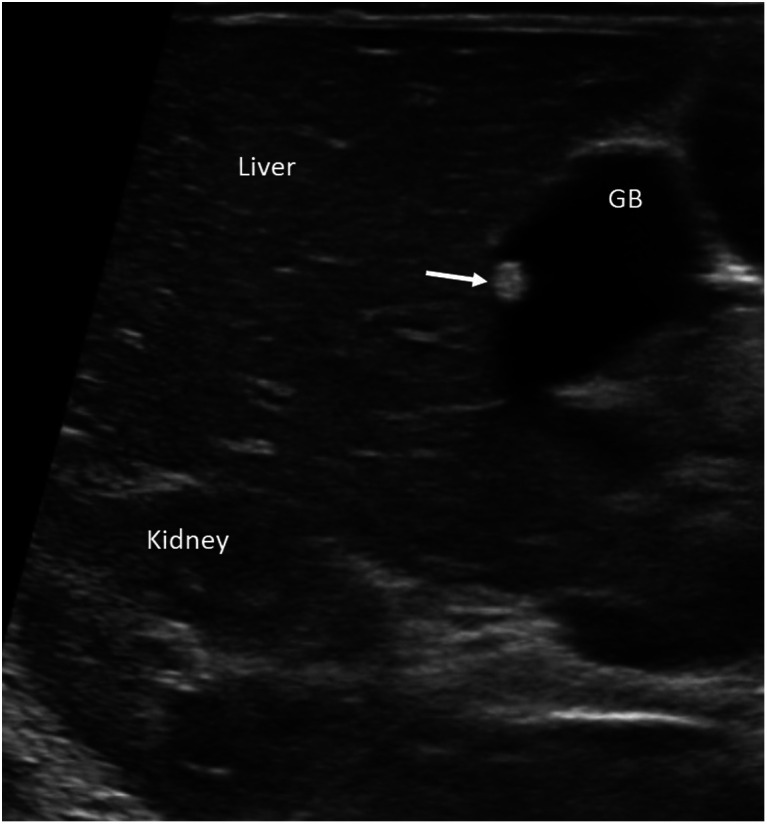
Transverse abdominal US of the gallbladder (case number 8) shows an echogenic polyp *(arrow)* in the gallbladder. GB, gallbladder; US, ultrasonography.

**Figure 2.**
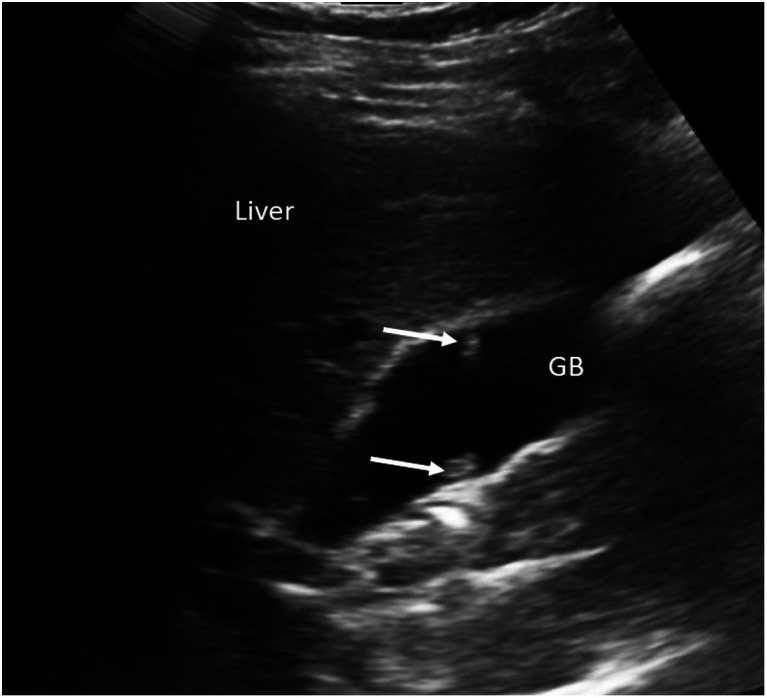
Longitudinal abdominal US of the gallbladder (case number 14) with multiple echogenic polyps with various diameters *(arrows)* in the gallbladder. GB, gallbladder; US, ultrasonography.

The records of 15 patients with a mean age of 13.2 years (2-20 years; 2 of the patients who are 19 and 20 years old during data collection were younger than 18 years old on admission) were included. Table 1 shows the demographic features, diagnostic characteristics, and follow-up characteristics of the patients. The male : female ratio was 7 : 8. Gallbladder polyp was detected incidentally in 73.3% (n = 11) of patients (Figure 1). Four of the patients were symptomatic (26.7%) and complained about abdominal pain at the right upper quadrant. Laboratory tests were normal except in 3 patients who showed slightly increased liver function tests. Two of the cases had normal gamma-glutamyl transferase (GGT) and aspartat aminotransferase (AST) levels on admission (case number 2 and 6) and their GGT and AST levels were slightly increased during the follow-up [patient 2; GGT: 90 IU/L (normal range: 0-30 IU/L), patient 3; AST: 66 IU/L (normal range; 0-40 IU/L)]. Only patient 12 had increased GGT and alanine aminotransferase levels on admission with clinical symptoms. Otherwise, GGT levels and other liver functions tests were within the normal limits in the rest of the patients. Two of the patients had 3 polyps on admission (Figure 2). The polyps were 2-10 mm in size. The size of the polyp increased in 5 patients (33.3%) and polyps disappeared in 4 patients (26.6%) in follow-up US. Five of the patients underwent cholecystectomy and 1 of them was scheduled for surgery. The indications of surgical treatment were polyps larger than 10 mm, polyps increased in size, and symptomatic patients regardless of size. Five of the asymptomatic patients who have polyps less than 10 mm are still on follow-up. In patients with cholecystectomy, the histopathology of gallbladders revealed cholesterol polyps (n = 2) and hyperplastic polyps (n = 2). One of the patients with cholecystectomy showed no polyps in pathology examination.

## Discussion

Despite multicenter studies, small cohorts of patients with GP have been reported in the pediatric population. Similar to the previous series, our results showed that GP might display different clinical courses in children.^1,2^ During the observational period, GP may either increase in size or may disappear. Surveillance with the US can be performed for asymptomatic patients with less than 10-mm polyps. Since progressive epithelial overgrowth of gallbladder mucosa is considered as a potential for malignant transformation, a close follow-up with US is recommended. No criteria have been established for the malignant potential of GP in children. Moreover, there is no clear algorithm for the treatment of GP in the pediatric population.

Most of the GPs are primary with unknown etiology. Hyperlipidemia is considered as a risk factor for the development of GP in both adults and children. Lee et al.^9^ found that low high-density lipoprotein, high low-density lipoprotein, and high levels of total cholesterol showed a strong correlation between GP development. However, no link has been established with gender, obesity, exogenous sex hormones, or hyperlipidemia in pediatric cases.^1,10^ In our series, serum lipids were within the normal limits in all patients including patients with cholesterol polyps.

Gallbladder polyps can be seen in all ages even in small infants. The median age of the patients varies from 10 to 13 years in different series.^1,2^ Although females are significantly outnumbering males, there was no sex predilection in the current series. The incidence of symptomatic cases is reported to be as high as 50% for pediatric cases.^2^ Incidental finding of a GP during US evaluation is more common in children and reported to be 79% in different series. It has been suggested that bile stones accompanying GP are associated with symptoms; therefore, symptomatic cases are more common in adults compared to children. In the current cohort of patients, we found that 73% of children were diagnosed with GP during the abdominal US for other causes of abdominal pain rather than biliary symptoms.

The common use of US in the diagnosis of gallbladder stones yield increased detection of GP in children. Ultrasonography has high sensitivity (80%) and specificity (99.3%) in the diagnosis of GP larger than 10 mm.^5^ Repeated US examinations are recommended for diagnosis. It has been reported that 94% of benign lesions are less than 10 mm and the polyp size is considered as a major determinant for malignancy.^11^ Csendes et al.^12^ performed the serial US in 98 asymptomatic adults with GPs less than 10 mm. The mean follow-up period of 6 years showed that half of the GP remain the same in size, whereas 25% increased and 25% decreased in size.^12^ They report no malignancy during the follow-up period. The differential diagnosis of GP includes gallbladder stones, sludge balls, and blood clots. Stones and blood clots move with postural changes whereas polyps are fixed.^13^ Also, polyps show echogenicity of soft tissue and do not have an acoustic shadow. During the US evaluation, evaluation of associated stones has paramount importance, because of risk assessment for malignant transformation.

Cholesterol polyps are the most common finding in histopathology and are reported in 70% of adult series.^14^ They are almost benign in nature and stable in size. Epithelial polyps are the second most common type and have tubular or papillary architecture. The latter type has a risk of malignancy especially in patients with larger polyps (more than 10 mm in size) or associating a gallbladder stone.^2^ In children, associated conditions such as metachromatic leucodystrophy, Peutz–Jegher Syndrome, or pancreaticobiliary malfunction increase the risk of malignancy. None of our patients had an associating anomaly. Two of the patients had a hyperplastic polyp in histology and underwent detailed evaluation for an underlying disease after cholecystectomy. Some of the polyps can disappear during the follow-up. Beck et al.^7^ reported that 2 of the pediatric cases showed no polyps in histopathology after cholecystectomy. Also, we found no polyps in pathology in 1 of the patients after 2 preoperative US. It has been recommended that at least 2 US should be performed for diagnosis.^1^ In our series, polyps disappeared in 4 of the cases during the follow-up. Therefore, we suggest that all patients should be re-evaluated by US before cholecystectomy to avoid unnecessary surgery since some of the polyps spontaneously disappear during the follow-up.

The treatment of GP in children depends on the symptoms of patients and the size of the polyps. Although asymptomatic patients with GP less than 10 mm can be followed up safely, cholecystectomy is the choice of treatment in larger polyps, multiple polyps, and symptomatic cases. Sessile and solitary polyps with irregular borders, presence of gallbladder stones, and thickening of gallbladder wall adjacent to GP are also accepted as risk factors for malignancy.^1^

Our study has some limitations. First, similar to previous ones, we report a small cohort of patients because of the rarity of GP in the pediatric population. Second, the study includes retrospective analysis and there is no standard treatment and follow-up algorithm for the patients. However, we perform cholecystectomy for the patients with multiple and larger polyps and for symptomatic cases. Since malignant transformation is extremely rare in children, the presence of large polyp as a surgical indication is well accepted by most pediatric surgical centers. Finally, there is no information about the histology of patients who are on US follow-up. Multicenter studies and meta-analysis evaluating the results of long-term follow-up of children with GP are needed to define the risk assessment of malignancy in children.

In conclusion, despite the lack of a standardized algorithm in children, our data suggested that multiple polyps, polyps with increased in size or greater than 10 mm, and the presence of symptoms might require cholecystectomy in children. Asymptomatic patients with small-sized polyps can be followed up with US and the polyps may disappear during the follow-up. Repeated US is mandatory to avoid unnecessary cholecystectomy in children.

## Figures and Tables

**Table 1.** Demographic Features, Diagnostic and Clinical Findings of Children with GP

No.	Age (year)	Gender	Symptoms	Laboratory Findings	Number and Size of Polyps at Diagnosis (n, mm)	Number of US During the Follow-Up	Number and Size of polyps at the Final Follow-Up (n, mm)	Management	Complication	Pathology	Duration of Follow-Up (months)
1	16	M	Incidental	N	3-6	3	1-7 mm	Follow-up	-	-	72
2	8	F	Incidental	GGT) ↑^*^	1-3	3	1-3.4 mm	Follow-up	-	-	30
3	10	F	Incidental	N	1-2	-	-	Follow-up	-	-	6
4	17	F	Incidental	N	1-4	4	1-6 mm	Follow-up	-	-	36
5	15	F	Incidental	N	1-6	4	1-5 mm	Follow-up	-	-	24
6	18	M	Incidental	AST ↑^*^	1-3	2	Disappeared	Follow-up	-	-	8
7	16	F	Incidental	N	1-5	4	Disappeared	Follow-up	-	-	24
8	2.5	F	Incidental	N	1-2.5	3	Disappeared	Follow-up	-	-	18
9	18	M	Incidental	N	1-2.5	5	Disappeared	Follow-up	-	-	36
10	19	F	Abdominal pain	N	1-3	4	2-6	Surgery	No	Cholesterol polyp	24
11	20	M	Abdominal pain	N	1-2.5	3	1-4	Surgery	No	Hyperplastic polyp	24
12	1.5	M	Abdominal pain, acholic stool	GGT ↑ ALT↑^**^	1-4	2	1-4	Surgery	No	No polyp	6
13	5	M	Incidental	N	1-5	2	1-5	Surgery	No	Cholesterol polyp	24
14	15	F	Abdominal pain	N	1-10	1	3-10	Surgery	No	Hyperplastic polyp	12
15	17	M	Incidental	N	1-5	3	3-5	Surgery planned	-	-	30

^*^Patients number 2 and 6 showed slightly increased GGT and AST levels during the follow-up. They were within normal levels on admission.

^**^Patients number 12 had increased levels of GGT and ALT on admission and remarkable increased levels with clinical symptoms.

M, male; F, female; N, normal; GGT, gamma-glutamyl transferase; ALT, alanine aminotransferase.
